# Tin Diselenide (SnSe_2_) Van der Waals Semiconductor: Surface Chemical Reactivity, Ambient Stability, Chemical and Optical Sensors

**DOI:** 10.3390/ma15031154

**Published:** 2022-02-02

**Authors:** Gianluca D’Olimpio, Daniel Farias, Chia-Nung Kuo, Luca Ottaviano, Chin Shan Lue, Danil W. Boukhvalov, Antonio Politano

**Affiliations:** 1 Department of Physical and Chemical Sciences, University of L’Aquila, via Vetoio, 67100 L’Aquila, Italy; gianluca.dolimpio@univaq.it (G.D.); luca.ottaviano@aquila.infn.it (L.O.); 2 Departamento de Física de la Materia Condensada, Universidad Autónoma de Madrid, 28049 Madrid, Spain; 3 Instituto “Nicolás Cabrera”, Universidad Autónoma de Madrid, 28049 Madrid, Spain; 4 Condensed Matter Physics Center (IFIMAC), 28049 Madrid, Spain; 5 Department of Physics, National Cheng Kung University, 1 Ta-Hsueh Road, Tainan 70101, Taiwan; kuochianung@gmail.com (C.-N.K.); cslue@mail.ncku.edu.tw (C.S.L.); 6 Taiwan Consortium of Emergent Crystalline Materials, Ministry of Science and Technology, Taipei 10601, Taiwan; 7 CNR-SPIN UoS L’Aquila, Via Vetoio, 67100 L’Aquila, Italy; 8 College of Science, Institute of Materials Physics and Chemistry, Nanjing Forestry University, Nanjing 210037, China; 9 Theoretical Physics and Applied Mathematics Department, Ural Federal University, Mira Street 19, 620002 Ekaterinburg, Russia; 10 CNR-IMM Istituto per la Microelettronica e Microsistemi, VIII strada 5, I-95121 Catania, Italy

**Keywords:** van der Waals semiconductors, gas sensing, tin diselenide, density functional theory

## Abstract

Tin diselenide (SnSe_2_) is a layered semiconductor with broad application capabilities in the fields of energy storage, photocatalysis, and photodetection. Here, we correlate the physicochemical properties of this van der Waals semiconductor to sensing applications for detecting chemical species (chemosensors) and millimeter waves (terahertz photodetectors) by combining experiments of high-resolution electron energy loss spectroscopy and X-ray photoelectron spectroscopy with density functional theory. The response of the pristine, defective, and oxidized SnSe_2_ surface towards H_2_, H_2_O, H_2_S, NH_3_, and NO_2_ analytes was investigated. Furthermore, the effects of the thickness were assessed for monolayer, bilayer, and bulk samples of SnSe_2_. The formation of a sub-nanometric SnO_2_ skin over the SnSe_2_ surface (self-assembled SnO_2_/SnSe_2_ heterostructure) corresponds to a strong adsorption of all analytes. The formation of non-covalent bonds between SnO_2_ and analytes corresponds to an increase of the magnitude of the transferred charge. The theoretical model nicely fits experimental data on gas response to analytes, validating the SnO_2_/SnSe_2_ heterostructure as a suitable playground for sensing of noxious gases, with sensitivities of 0.43, 2.13, 0.11, 1.06 [ppm]^−1^ for H_2_, H_2_S, NH_3_, and NO_2,_ respectively. The corresponding limit of detection is 5 ppm, 10 ppb, 250 ppb, and 400 ppb for H_2_, H_2_S, NH_3_, and NO_2,_ respectively. Furthermore, SnSe_2_-based sensors are also suitable for fast large-area imaging applications at room temperature for millimeter waves in the THz range.

## 1. Introduction

Following the advent of graphene [1,2,3,4], the scientific community has begun to consider layered semiconductors for their potential application to complement those of graphene [5,6,7,8,9], thus generating promising new technologies in various technological areas [10,11,12,13,14,15,16]. The class of materials of ‘van der Waals semiconductors’ is characterized by weak van der Waals bonds between the layers that allow their exfoliation by mechanical [17,18] and liquid-phase [19,20,21] exfoliation.

The most common van der Waals semiconductors have shown limitations, which compromise their technological development. For example, MoS_2_ and WS_2_ have a poor electric mobility of a few tens of cm^2^V^−1^s^−1^ at T = 300 K [22]; black phosphorus is instable in air and undergoes a rapid surface oxidation which degrades the morphology of the surface [23]; GaSe exhibits instability upon both laser and air exposure [24,25]; and PdSe_2_ [26,27] has a limited commercial potential, due to the constantly growing price of Pd (2000–2400 $/oz), nearly doubled in 2019–2021.

**Figure 1:**
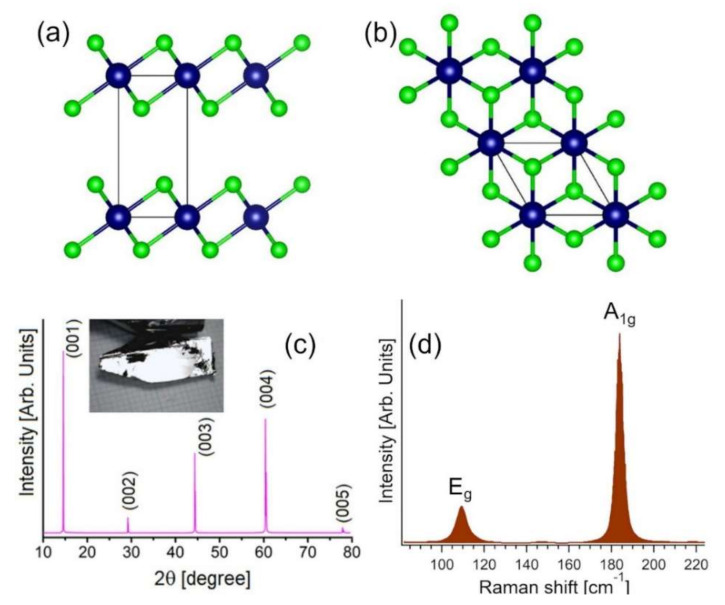
(**a**) Side and (**b**) top views of the atomic structure of SnSe_2_. Green and blue balls denote Se and Sn atoms, respectively. Panel (**c**) reports the single-crystal XRD pattern from the (001) plane of SnSe_2_. The inset shows a photograph of an as-grown SnSe_2_ single crystal. Panel (**d**) reports the Raman spectrum of SnSe_2_ single crystal acquired at room temperature with a laser with wavelength λ = 632.8 nm.

Tin diselenide (SnSe_2_) is a layered semiconductor constituted by Earth-abundant and cheap elements [28], which crystallizes in a layered CdI_2_–type structure with hexagonally packed layers of Sn atoms sandwiched between two layers of Se anions (Figure 1a,b) [29,30,31]. Differently from MoS_2_ and WS_2_, SnSe_2_ has a high intrinsic electron mobility at T = 300 K (462.6 cm^2^V^−1^s^−1^) and ultralow thermal conductivity (3.82 W m^−1^ K^−1^) [32]. Furthermore, SnSe_2_ exhibits pressure-induced periodic lattice distortion and, moreover its atomic structure can reversibly change from amorphous to crystalline upon laser heating, being a phase change memory material. Owing to these peculiarities, SnSe_2_ has high application capabilities in several fields, including superconductivity [33,34], Li^−^ [29,35] and Na^−^ [29,36] ion batteries, photodetection [37], photocatalysis [38,39], saturable absorbers for eye-safe lasers [40], and thermoelectricity [41,42]. 

Nevertheless, Sn-based chalcogenides suffer from rapid surface oxidation with the formation of surface tin-oxide phases [43,44]. Furthermore, during the crystal synthesis process, tin could already oxidize, modifying its overall transport properties [45]. Therefore, the use of Sn-based chalcogenides for technology transfer remains particularly arduous. Especially, stability in ambient atmosphere of SnSe_2_-based devices is related to the chemical reactivity of its surface.

Here, we unveil surface properties of SnSe_2_ single crystals by means of surface-science experiments and density functional theory (DFT). We demonstrate that the stoichiometric SnSe_2_ sample is chemically inert, while the presence of Se vacancies induces surface oxidation with the formation of a sub-nanometric SnO_2_ skin. We also explore the capability of SnSe_2_ to realize devices for sensors for detecting noxious gases and imaging applications with non-ionizing radiations. Especially, we show that chemical sensing is feasible only when the pristine SnSe_2_ surface is transformed into an heterostructure of SnO_2_/SnSe_2_. Concerning photodetection, we report the design of broadband SnSe_2_-based photodetectors interplayed by synergistic effects of multiple mechanisms. Considerably, the effect of hot electrons in ultrashort channel devices under strong light coupling results in outstanding performance in term of high responsivity at THz frequency. 

## 2. Materials and Methods

Single crystals of SnSe_2_ were grown by Bridgman–Stockbarger (Figure 1c). Stoichiometric ratio of 1:2 was put on evacuated quartz ampoule. The growth was carried out in a vertical two-zone tube furnace. The obtained crystal was characterized with X-ray diffraction (XRD) on powders, as shown Figure 1c. From the XRD spectrum, we can conclude that the crystal structure is CdI_2_–type (space group P-3m1). The lattice parameters are a = 0.3804 nm and c = 0.6128 nm consistently with previous works [46,47,48,49]. We also carried out the XRD and Laue diffraction measurements on single crystals. Samples were exfoliated in situ by adhesive tape. The absence of contamination in grown single crystals is secured by the survey X-ray photoelectron spectroscopy (XPS) spectrum.

XPS experiments were carried out with synchrotron light at APE-HE beamline at the Elettra Synchrotron in Trieste, Italy.

High resolution electron energy loss spectroscopy (HREELS) experiments were performed with a Delta 0.5 spectrometer (Specs GmbH, Germany). Spectra were taken in specular geometry, with an impinging angle of 55° with respect to the perpendicular direction to the surface. The impinging energy is 3.5 eV.

Theoretical methods are reported in Section S1 of the Supplementary Materials.

Fabrication process and measurements of devices are reported in section S2 of the Supplementary Materials.

## 3. Results

### 3.1. Chemisorption of O_2_ and H_2_O on Bulk SnSe_2_

The Raman spectrum of the grown SnSe_2_ single crystal (Figure 1d) shows the E_g_ and A_1g_ modes at 109 and 184 cm^−1^, respectively, congruently with previous reports [50,51,52]. The narrow (00l) diffraction peaks (Figure 1c) reveal the excellent crystallinity for our SnSe_2_ crystals.

**Figure 2:**
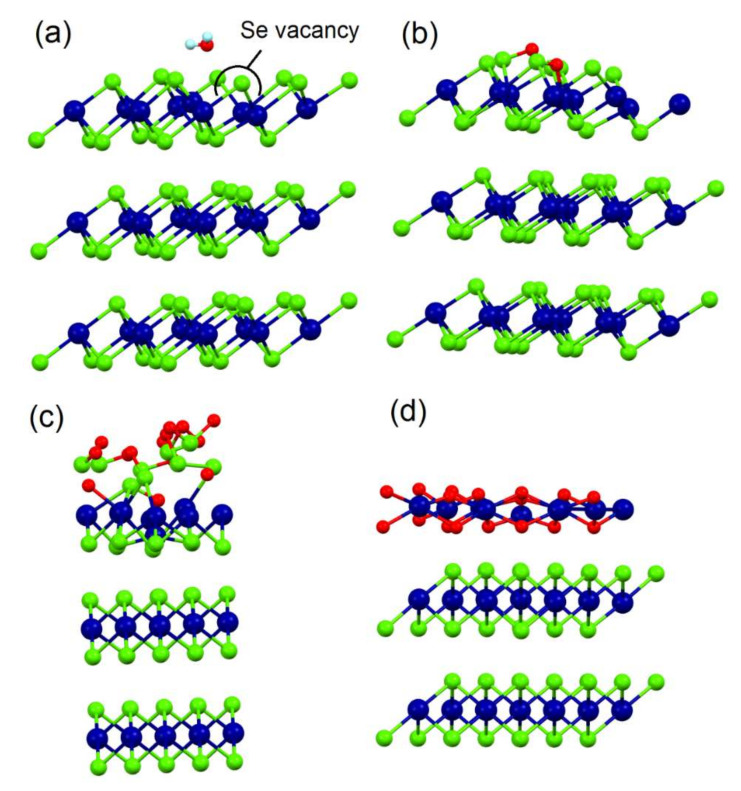
Optimized atomic structure of (**a**) water molecules physisorbed at one Se-vacancy site; (**b**) decomposed oxygen molecule on SnSe surface layer; (**c**) metastable SnSe_2_O_2_ surface layer; and (**d**) SnO_2_-skin-terminated SnSe_2_.

To model surface chemical reactivity, the differential enthalpy ΔH_ads_ and the differential Gibbs free energy ΔG for the adsorption of water and oxygen at room temperature, as well as the decomposition energy ΔH_dec_ for both gases were calculated. The possibility of different kinds of defects was energetically evaluated and the formation of one Se vacancy is particularly feasible (only 1.28 eV/Se). The influence of Se vacancies was explored from a single vacancy in the outermost surface layer (Figure 2a) up to larger concentration of Se vacancies. We also calculated the different possible positions for a second Se vacancy, finding that the most energetically favorable location (0.98 eV/Se) is to have the second vacancy in the next neighbor to the first vacancy. To model such a large number of vacancies, an outermost SnSe-like layer on the surface was also considered (Figure 2b). Calculations indicate that physisorption of molecular oxygen is feasible at all investigated surfaces, although it is more energetically favorable at Se vacancies (ΔG = −26.3 kJ/mol) rather than on defects-free SnSe_2_ (ΔG = −3.2 kJ/mol). The subsequent decomposition of molecular oxygen is an exothermic process for SnSe_2_, SnSe_1.88_, and SnSe. However, in the case of SnSe_1.88_ and SnSe the differential enthalpy of decomposition is much lower than the defect-free SnSe_2_ (−135.7, −236.1, and −42.3 kJ/mol, respectively). Thus, the oxidation rate should be greater on defective surfaces of SnSe_2_. After the decomposition of a single oxygen molecule, we assess the oxygenation of the whole surface, corresponding to an atomic structure with oxygen atom attached to each surface Se atom, and the successive oxidation in a metastable surface SnSe_2_O_2_ phase (see Figure 2c), whose lifetime is estimated to be < 1 ms and, consequently, its presence on the surface could be detected only with time-resolved experiments. Hence, first the Se atoms in the SnSe_2_O_2_ migrate to occupy the Se vacancies formed in the subsurface region and then the oxygen atoms from the SnSe_2_O_2_ oxidize the Sn atoms of the surface layer to form SnO_2_, see Figure 2d. The following chemical equation can describe this process as

O_2_ + SnSe_2_ + SnSe_2-y_ → SnSe_2_O_2_ + SnSe_2-y_ → SnO_2_ + 2Se + SnSe_2-y_ → SnO_2_ + SnSe_2-x_ (1)

with x < y, where SnSe_2-y_ corresponds to Se defects in the substrate, which are partially or totally saturated by Se freed from top layer after formation of SnO_2_-skin.

We calculated the necessary energy to heal the Se vacancy by extracting Se from the SnO_2_ skin and the differential enthalpy for this reaction is −3.54 eV/Se for the oxidized surface of SnSe_2_ and −2.97 eV/Se for oxidized surface of SnSe_1.88_. These values cause a preferential oxidation of Sn with respect to Se and, subsequently, the surface oxide layer should be modelled as a SnO_2_/SnSe_2_ heterostructure.

Instead, the physisorption of water is energetically unfavorable on SnSe_2_. Although near the Se vacancy the energy barrier for water absorption decreases it always remains a metastable process with positive ΔG in the Se vacancy sites (ΔG = +3.4 kJ/mol). Even in the defect sites, a subsequent decomposition of water is extremely unfavorable (ΔH_dec_ = +175.7 kJ/mol). This behavior can be understood because the water molecules interact with diselenides via the formation of non-covalent bonds between lone pairs of electrons on *sp* orbitals of oxygen and unoccupied orbitals of metal centers in substrate. In the case of SnSe_2_, some mismatch between the size of water molecule and lattice parameters of the substrate is not so favorable for the formation of the described non-covalent bonds. The contribution to the adsorption energy calculated from the energy cost of the substrate and molecule distortions decreases ΔH, making it lower than the TΔS contribution in the ΔG calculation. In the case of adsorption on an oxidized substrate, hydrogen bonds are established between water and substrate oxygen. The formation of these hydrogen bonds can occur at much broader range of positions of water on the substrate and, therefore, no contribution in ΔH comes from distortions of either substrate or water.

### 3.2. Experimental Validation of the Theoretical Model

**Figure 3:**
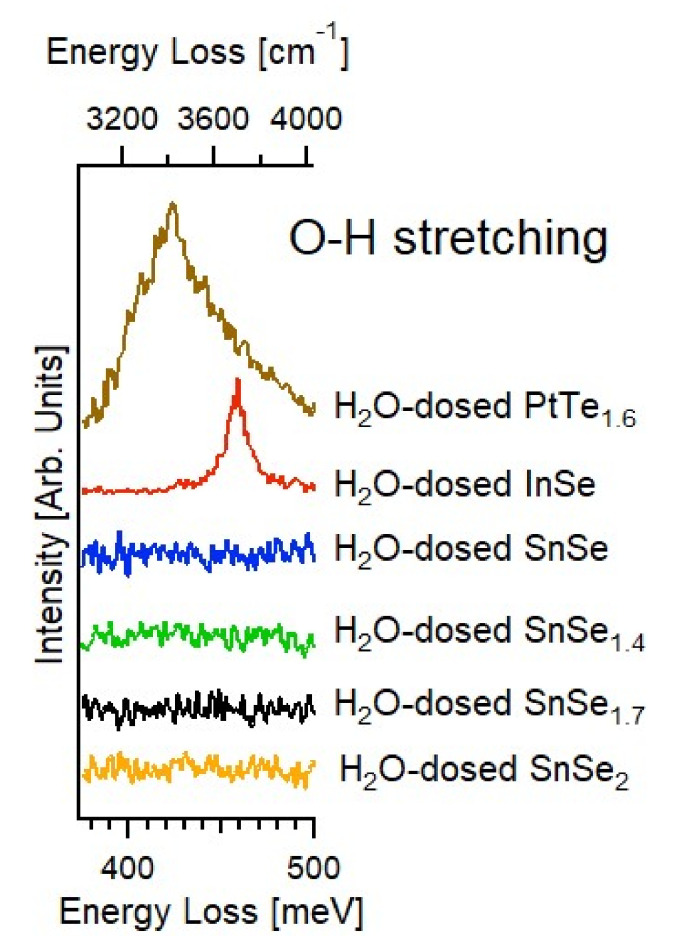
Vibrational spectra around the energy of O-H intramolecular stretching, recorded upon dosing to 10^5^ L of H_2_O at T = 300 K the surfaces of various tin-based selenides: SnSe_2_ (orange), SnSe_1.7_ (black), SnSe_1.4_ (green), and SnSe (blue). We also report vibrational acquired in the same conditions for water-dosed InSe (red) and PtTe_1.6_ (brown). The energy of the primary electron beam is 4 eV.

Probing vibrational modes could afford additional information on surface chemical processes and, especially, physicochemical mechanisms ruling the formation of an oxide skin. In particular, high-resolution electron energy loss spectroscopy (HREELS) experiments on H_2_O-exposed tin selenides SnSex, with x ranging between 1 and 2 (SnSe, SnSe_1.4_, SnSe_1.7_, SnSe_2_), indicate the lack of chemisorbed molecules resulting from the presence of H_2_O, this is evident from the absence of O-H streching at 410–420 meV (molecular water) and 450 meV (hydroxyl groups) in the spectra in Figure 3 [53]. These findings are consistent with the positive Gibbs free energy of adsorption (corresponding to energetically unfavorable water adsorption) in Table 1. For a better comparison, we report in Figure 3 the vibrational data obtained by exposing other chalcogenides to the same dose of water (10^5^ L). Unlike the surface of SnSex, a stable adsorption of water molecules was found on PtTe_1.6_ and, moreover, of hydroxyl groups on InSe. The absence of reactivity toward water of Sn-based chalcogenides makes them suitable for catalysis (in particular, photocatalytic water splitting [38], and hydrogen evolution reaction [54]) and drug delivery [55] (also bearing in mind that neither Sn nor Se are toxic).

**Figure 4:**
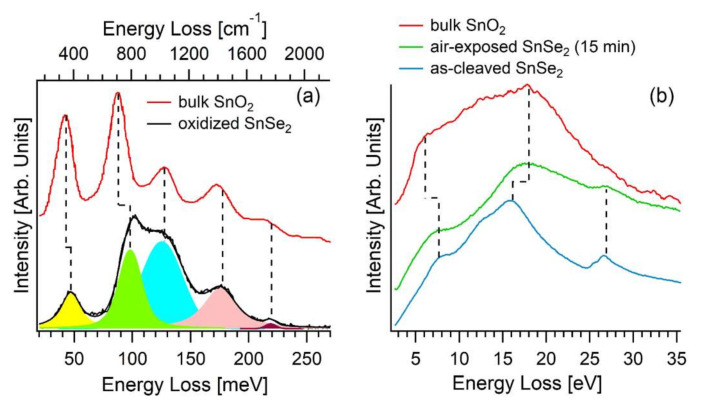
(**a**) Vibrational data for oxidized SnSe_2_(001) (recorded by HREELS with impinging energy of 4 eV) and bulk SnO_2_(110) (data taken from [56]). (**b**) Excitation spectrum for pristine and air-modified SnSe_2_ (recorded by EELS with impinging energy of 300 eV) and bulk SnO_2_ (data taken from [57]).

Notably, the vibrational spectrum of the oxidized SnSe_2_ surface (Figure 4a) closely overlaps with the phonon excitation spectrum of SnO_2_ [56]. Definitely, modes at 48, 99, 126, 177, and 219 meV were measured. In particular, the loss peaks at 48 (A_2g_ phonon) and 99 (B_2g_ phonon) meV are blue-shifted by 4 and 10 meV in the disordered tin-oxide skin formed upon oxidation of SnSe_2_ compared to their respective value for bulk SnO_2_ crystals [56]. 

**Figure 5:**
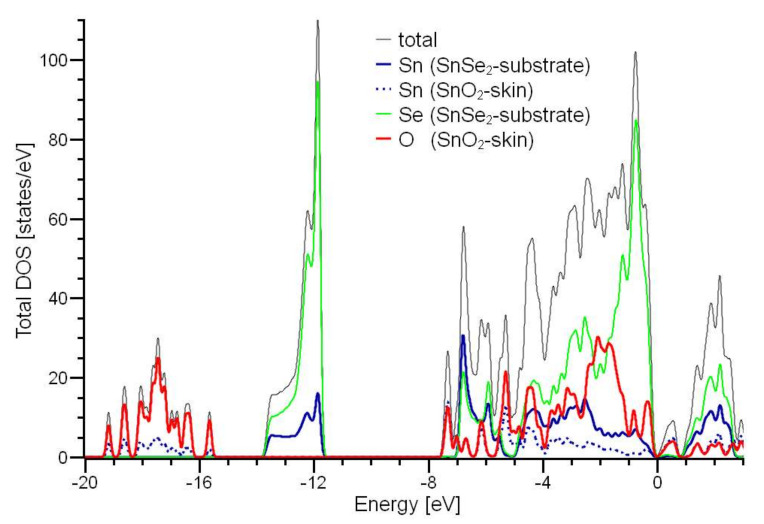
Partial densities of states for SnSe_2_ slab with SnO_2_-skin (see Figure 2d). Fermi energy is set as zero.

The inspection of the excitation spectrum probed by EELS, extended up to the ultraviolet range of the electromagnetic spectrum (Figure 4b), enables monitoring the surface status with a technique with probing depth as low as (0.9 ± 0.1) nm in our experimental conditions [58], which is lower by more than 10^2^ with respect to Raman spectroscopy and optical techniques. Specifically, the excitation spectrum of the as-cleaved SnSe_2_ surface shows a main feature at 15.9 eV with a shoulder at 12.0 eV, ascribed to interband transitions from Se-4s core levels and, moreover, two weak losses at 7.5 and 26.8 eV. The excitation spectrum of the air-exposed SnSe_2_ sample is dominated by an emerging broad mode centered around ~18 eV, with two weak peaks at 7.5 and 26.8 eV, evidently insensitive to surface modification. Notably, polycrystalline SnO_2_ films display the feature at 18.0 eV. Precisely, this feature was previously attributed to the sub-oxide SnO_2-x_ phases [59]. However, the inspection of density of states (DOS) in Figure 5 reveals that the mode at 18.0 eV is related to a single-particle transition starting from O-2s band in SnO_2_. The weak peaks at 7.5 and 26.8 eV are ascribed to interband transitions originated by Sn-5*s* and Se-3*s* levels, respectively. 

**Figure 6:**
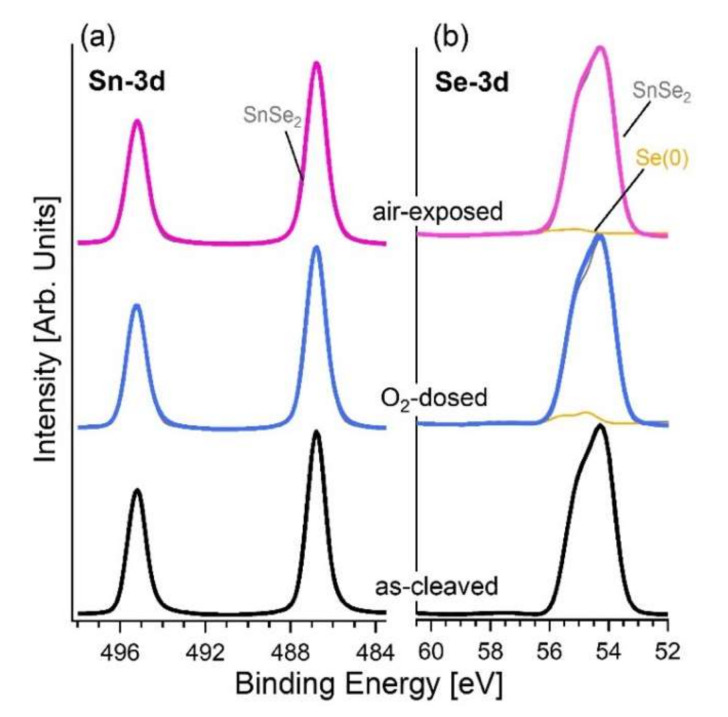
Panels (**a**) and (**b**) show Sn-3d and Se-3d core levels for as-cleaved surface of SnSe_2_ and its modification upon O_2_ (10^5^ L) dosage and air exposure. The photon energy is 800 eV.

Further information on the surface properties was provided by the inspection of core levels by means XPS experiments performed on both pristine and powderized SnSe_2_ single crystals. As a matter of fact, it is also important to assess the characteristics of the powderized material with the future implementation in devices in mind, for which the surface/volume ratio should be maximized in order to improve the performance. Figure 6 shows the Sn-3d and Se-3d core levels of the as-cleaved SnSe_2_ single-crystal surface and for the same surface modified by O_2_ dosage with a total dose of 10^5^ L (1 L = 10^-6^ Torr·s). The Sn-3d_5/2_ core level of the as-cleaved sample has a binding energy (BE) of 486.8 eV (Figure 6a). Correspondingly, the Se-3d core levels exhibit a single peak with the J = 5/2 component located at BE = 54.1 eV, compatibly with previous reports for SnSe_2_ [60] and with a lower BE compared to the case of SnSe (BE = 53.7 eV). Exposure to 10^5^ L of oxygen and storage in air only caused a slight change in the core level of the Se-3d. A new component at BE = 54.7 eV in Se-3d core level arising from Se(0) segregation is observed [61]. The total spectral area of this new component is 5.4% for O_2_ dosage and 2.6% for air exposure. Especially, from the Se-3d core-level spectra (Figure 6b) one can observe the absence of the SnO_2_ component, which would be characterized by the J = 5/2 component at BE of ~59–60 eV.

**Figure 7:**
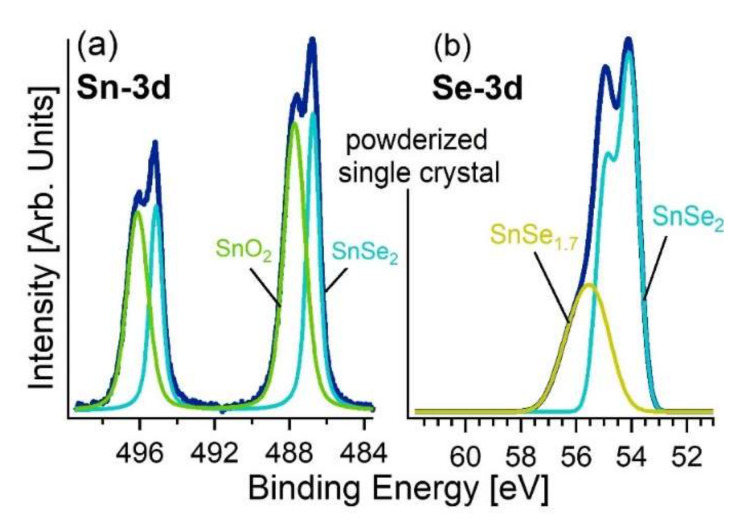
(**a**) Sn-3d and (**b**) Se-3d core levels of powderized SnSe_2_ single crystal. Note that grinding of SnSe_2_ bulk crystals was carried out in ambient atmosphere. The photon energy is 1486.6 eV (Al K_α_).

Conversely, in the powderized single crystal, one spectral component arising from surface oxidation was found (Figure 7). Specifically, the new component in the Sn-3d_5/2_ core level related to SnO_2_ was observed at BE = 487.8 eV (54% of the total spectral area) [62,63]. Remarkably, even after powderization, no trace of SeO_2_ is present, as indicated by the featureless Se-3d spectra in the range 59–60 eV [64]. This finding confirms our prediction that Se is only involved in a metastable oxide phase, which represents a precursor for SnO_2_ formation. However, in the powderized sample, a different Se oxidation state is present, as revealed by a broad feature in the spectra. Precisely, we assign the component at 55.0 eV to Se^−2^ and the higher to Se^−2+δ^ (0 < δ < 1) [65]. In the powderized sample, we estimate δ to be ~0.15 ± 0.05 from the analysis of the survey XPS spectrum. Therefore, sub-stoichiometric SnSe_1.7±0.1_ coexists with SnSe_2_. We also estimated the thickness of the SnO_2_ surface layer by means of quantitative analysis of XPS data [66], finding a thickness of (0.8 ± 0.1) nm (~2.5 monolayers) without observable changes after an exposure of one week in air. It should be noted that previous reports indicated that the surface of sub-stoichiometric SnSe_2_ (SnSe_1.71_) grown by molecular beam epitaxy [67] is unstable, with the subsequent formation of SnO_x_ and SeO_x_ phases. Conversely, in our case, both SnO and SeO_x_ are not present on the surface.

**Figure 8:**
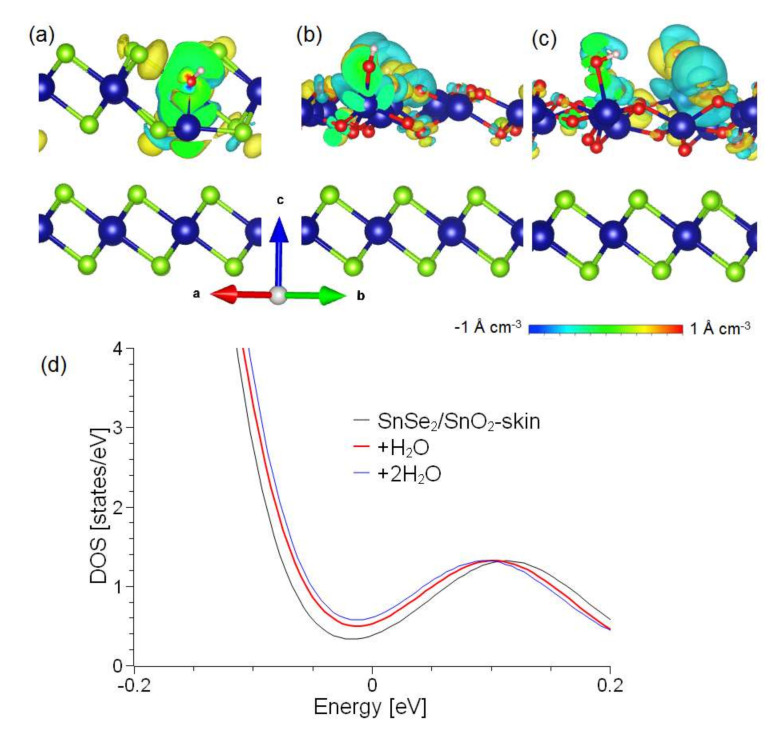
Panels (**a**-**c**) report the change of the charge density after adsorption of one water molecule on SnSe_2_, one water molecule on SnO_2_-skin-terminated SnSe_2_, and two water molecules on SnO_2_-skin-terminated SnSe_2_, respectively. Panel (**d**) represents the DOS of SnO_2_-skin-terminated SnSe_2_ (black curve) and of the same system modified by the adsorption of one (red curve) and two (blue curve) water molecules. Fermi level is set at 0.

The SnO_2_/SnSe_2_ heterostructure is more sensitive to chemisorbed species with respect to pristine SnSe_2_. On the pristine surface, the absorption of water molecules generates local changes in charge density in proximity to the absorbed molecules on the surface layer due to a rearrangement of chemical bonds, with a charge transfer of 0.17 e- per water molecule (Figure 8a). Thus, we conclude that pristine SnSe_2_ is unsuitable for humidity sensing. On the other hand, the absorption of H_2_O on SnO_2_/SnSe_2_ is energetically favorable even above room temperature. We calculated the values of transferred charge from H_2_O to the SnO_2_ skin that are 0.43 and 0.30 e^−^ for one and two H_2_O molecules per supercell, respectively. Correspondingly, the density of states (DOS) is modified with a direct correlation with the coverage of the adsorbate (Figure 8b–d), thus indicating the suitability for humidity sensing, even at the lowest concentrations of H_2_O. Note that decomposition of water molecule on the SnO_2_/SnSe_2_ heterostructure is an exothermic process (−121.4 kJ/mol) and the subsequent water splitting is unfavorable, favoring the reversibility of the process. This further supports the use of the self-assembled SnO_2_/SnSe_2_ heterostructure for humidity sensing.

### 3.3. Gas Sensing

The evaluation of the stability of SnSe_2_-based systems at the temperature of 200 °C, used in a typical gas sensing experiment, was performed. For the surface model of bulk SnSe_2,_ trilayers of SnSe_2_ with fixed lattice parameters were used. For free-standing bi- and monolayer optimization of both atomic position and lattice parameters was performed. This provides the contribution in the energetics of the adsorption from flexibility of free-standing few-layers. In order to make calculations more realistic, we also considered the presence of the Se vacancies in the top layer. The formation of the single vacancy turns the SnSe_2_ to SnSe_1.88_. Results of the calculations (see Table 2) demonstrate that physical adsorption on bulk and few-layers of SnSe_2_ is unfavorable at 200 °C. Contrarily, the presence of Se-vacancies makes physisorption favorable. The decomposition of molecular oxygen is favorable in all cases except for monolayer SnSe_1.88_. This exception is due to the combination of distortions caused by the presence of vacancies and distortions created by formation of new Se-O chemical bonds. Note that this result is valid only for free-standing monolayers. In fact, the deposition of a free-standing monolayer on a substrate decreases the flexibility of SnSe_x_ and makes the system closer to a SnSe_x_ bilayer.

The favorability of decomposition of oxygen molecule even at defects-free substrate of SnSe_2_ could be a starting point for a possible oxidation in the defective areas of SnSe_2_ (vacancies, edges, grain boundaries). The formation of SnO_2_ skin on the surface of bulk SnSe_2_ leads to a migration of Se-atoms to subsurface area with the passivation of Se-vacancies in the sub-surface layers [68]. In the case of free-standing few layers of SnSe_x_, an unstable structure would be formed. Therefore, we exclude this configuration from further investigation. Precisely, the discussed unstable structure is related to few free-standing layers, but if we include the presence of the substrate, the formation of more ordered SnO_2_ skin will occur. Thus, results obtained for SnSe_2_/SnO_2_ could be extended to few layers of SnSe_2_ deposited on a substrate.

**Figure 9:**
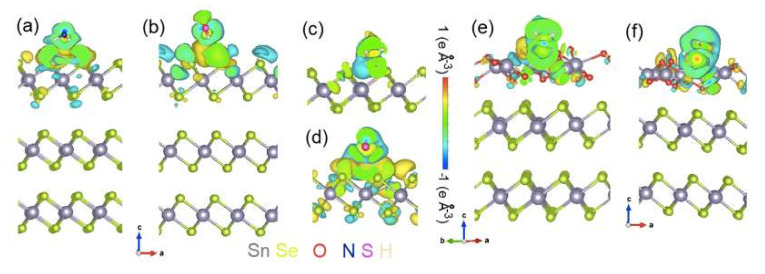
Change of charge densities after adsorption of (**a**,**c**,**e**) NH_3_ and (**b**,**d**,**f**) H_2_S on (**a**,**b**) bulk and (**c**,**d**) monolayer of SnSe_2_ and, moreover, (**e**,**f**) the SnO_2_ skin on an underlying SnSe_2_ substrate.

Considering the possibility of the oxidation with the formation of a SnO_2_ skin, we modeled the physisorption of different analytes on SnSe_x_ and on the SnO_2_ skin over SnSe_2_ substrate (Figure 9). Results of the calculations (see Table 3) demonstrate that the adsorption of various molecules on SnSe_x_ surfaces obeys the same principles as in the case of oxygen. Definitely, defect-free SnSe_2_ surface is nearly inert for all considered analytes. The presence of Se vacancies turns the free energy in a negative value making the adsorption favorable. Similarly, the adsorption of all analytes on monolayer SnSe_x_ is less favorable than the case of bilayer and bulk. In contrast to non-oxidized SnSe_x_ substrates, the formation of SnO_2_-skin corresponds to a strong adsorption of all analytes. The formation of non-covalent bonds between SnO_2_-substrate and analytes corresponds to an increase of magnitude of the transferred charge (see Figure 8). Note that the magnitudes of the free energies of adsorption and transferred charges in the case of SnO_2_ skin are much larger than in the case of adsorption on Se-vacancies (SnSe_1.88_ surfaces). Additionally, in the case of the adsorption on the SnO_2_ skin, the value of the transferred charge is strongly distinct for different analytes. 

**Figure 10:**
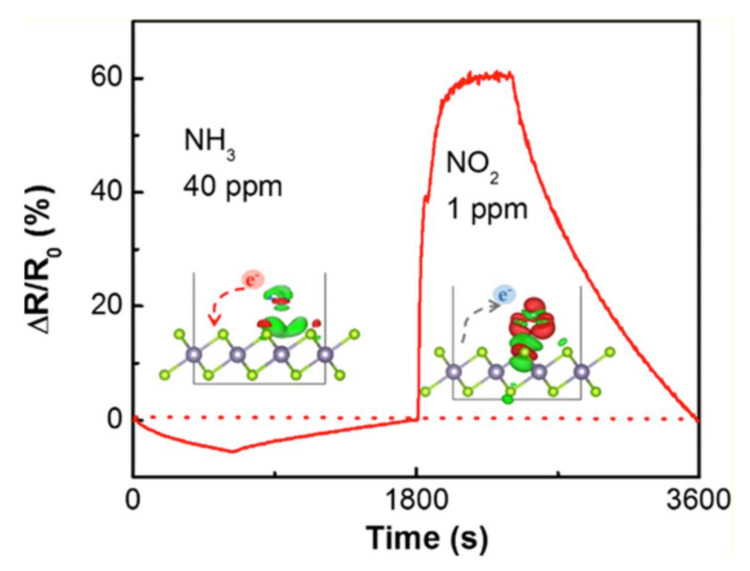
Dynamic response-recovery curve for NO_2_ (1 ppm) and NH_3_ (40 ppm). The inset depicts that electronic charge transfer occurs from SnSe_2_ to NO_2_, while the opposite charge transfer exists in the case of NH_3_, congruently with experimental findings on electrical tests. Reproduced with permission from [79].

The theoretical model discussed above was confirmed by experiments. The sensing properties of SnO_2_/SnSe_2_ heterostructures were measured by monitoring the resistance change upon exposure to different concentrations of gases. In Figure 10, the dynamic response-recovery curve with NH_3_ (40 ppm) and NO_2_ (1 ppm) is shown, with opposite signs in the response curve related to the opposite charge transfer, as depicted in the inset. Interestingly, sub-ppm limit of detection is feasible for both cases, with 250 and 400 ppb for NH_3_ and NO_2_, with near-room-temperature operational temperatures.

The SnO_2_/SnSe_2_ heterostructure exhibit significantly enhanced response and superb response/recovery characteristics, also for H_2_S, for which the lowest detection limit reaches 10 ppb with a response value of 2, which is below the acceptable ambient levels of 20–100 ppb set by the Scientific Advisory Board on Toxic Air Pollutants (USA) [69,70].

Similarly, H_2_ detection is feasible using SnO_2_/SnSe_2_ heterostructure [68] with a response of 3 with an operational temperature of 150 °C at a concentration of 100 ppm and a limit of detection of 5 ppm. Considering the quite reduced costs of raw elements, this could be relevant considering that in the growing market for H_2_-powered devices—i.e., fuel cells—there is a requirement for cheap H_2_ sensors. 

Table 4 reports an overview of literature results on near-room-temperature sensing of H_2_, H_2_S, NH_3_, and NO_2_ for SnSe_2_- and SnO_2_-based sensors, at their respective operational temperature and concentration. Sensitivities of 0.43, 2.13, 0.11, and 1.06 [ppm]^−1^ were reported for H_2_, H_2_S, NH_3_, and NO_2,_ respectively (Table 5, also reporting the limit of detection). 

The SnO_2_/SnSe_2_ heterostructure exhibits higher performance in terms of high response to analytes, low detection limit, high selectivity, and low power consumption [69,70,71,72,73,74]. Therefore, based on both calculations and experiments, one can conclude that the SnO_2_/SnSe_2_ heterostructure is a promising platform for gas sensing.

### 3.4. SnSe_2_-Based Sensors for Large-Area Imaging with Millimetre Waves 

**Figure 11:**
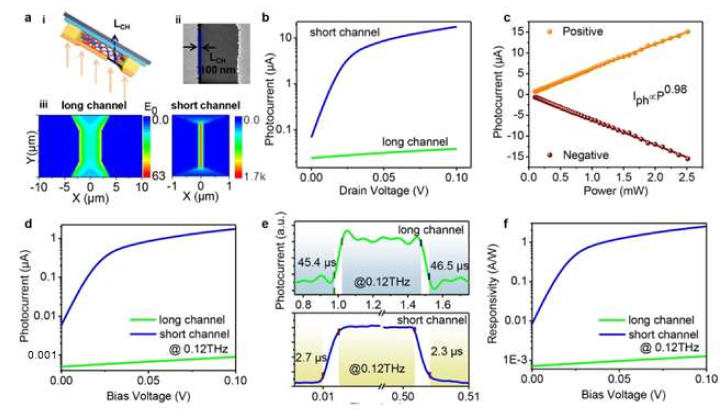
(**a**i) Schematization of the realization of the short-channel device. (**a**ii) Image of a short channel SnSe_2_-based photodetector acquired with the SEM. (**a**iii) The electric field distribution of the SnSe_2_-based long channel (left panel) and short channel (right panel) photodetector. (**b**) and (**c**) Photocurrent as a function of the bias voltage and incident power at 0.04 THz, respectively. (**d**) Photocurrent as a function of bias voltage at 0.12 THz. (**e**) (top panel) Long channel and (bottom panel) short channel time-resolved photocurrent devices at 0.12 THz with 1 mW cm^−2^ power. (**f**) Photoresponsivity vs. bias voltage at 0.12 THz with 1 mW cm^−2^ power. Reproduced with permission from [91].

An efficient THz detector at room temperature needs two characteristics: high mobility and a channel with nanometer length in a FET. Alternatively, the electron heat-driven detection mechanism could be used [86], based on the specific geometry of the device and the thermal/electrical properties of the material. In fact, SnSe_2_ has both a high thermoelectric power and a suitable bandgap [87], which are advantageous characteristics for the production of hot electrons and for photothermal conversion. To improve the THz photodetection performance of the SnSe_2_-based device, the relationship between channel length and photoresponse should be investigated. In FETs, an important issue concerning THz detection is that the channel length should be less than tens of nanometers at the expense of rectification ratio. As mentioned above, it is possible to exploit hot electrons to overcome this limit, related to the absorption efficiency of photons. Specifically, the performance of SnSe_2_-based THz detectors with a channel length of 6 µm and 100 nm, along with the simulated electric field intensity, were evaluated (Figure 11a). A short channel SnSe_2_ device is reported in Figure 11a-i,ii. In Figure 11a-iii, one can notice how the reduction of the antenna gap increases the value of the square of the electric field intensity (E_0_^2^) in the channel by almost three orders of magnitude. To study the different photoelectric properties of the devices as a function of the channel length, the photocurrent, the response time, and the reactivity were measured simultaneously using two radiations 0.04 and 0.12 THz with a power density of 2.5 and 1 mW cm^−2^, respectively. The photocurrent increases linearly under electrical bias as V_DS_ from −0.1 to 0.1 (Figure 11b), due to the increased carrier drift-velocity and reduced carrier transit time. Moreover, the short channel devices show a photocurrent greater than three orders of magnitude at 0.04 THz, congruently with the theoretical predictions of Figure 11a-iii. By exploiting the antenna gap in short channel device, it is possible to concentrate the THz radiation in a very small spot, consequently improving the SnSe_2_ intraband absorption capacity, thus hot carriers can be efficiently produced [88]. In Figure 11c, one can observe a good linearity over a wide range of the photocurrent, of the short channel device, as a function of the incident power. Using the equation I_ph_ ∝ P^β^ to fit the experimental data, β is ~0.98 under positive or negative bias, which is an interesting value for high-contrast imaging. Even at the frequency of 0.12 THz, there is the same improvement effect, as evident from Figure 11d, confirming the broadband nature of THz field nano-focusing. Additionally, the response time at 0.12 THz for both short and long channel devices was also studied (Figure 11e). For the short channel device, τ_rise_ =2.7µs and τ_fall_ = 2.3 µs—i.e., a response time of approximately 16 times faster than in the long channel device (τ_rise_ = 45.4 µs, τ_fall_ = 46.5 µs). Obviously, the long channel device has a slower response time than the short channel device, making the short channel device advantageous in applications. The increase in V_DS_ rapidly increases the bias voltage response to 0.12 THz (Figure 11f). For short channel devices, the maximum response is 2.5 A W^−1^, which is approximately five times larger than graphene-based devices (20 V W^−1^) [89] and 12 times larger than black-phosphorus-based devices (7.8 V W^−1^) [90]. This indicates that short-channel SnSe_2_ devices have superior performance respect to graphene- and black phosphorus-based devices.

**Figure 12:**
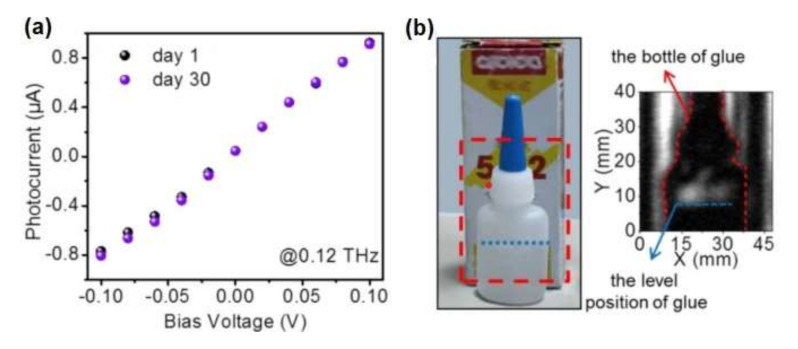
(**a**) Aging in air of SnSe_2_-based short channel photodetector, black and purple spot represent the photocurrent of as-prepared device and after 30 days in air, respectively. (**b**) The THz imaging of a glue-jar in the box at 0.12 THz. Reproduced with permission from [91].

Exploiting the excellent performance of the SnSe_2_, a large area imaging device with THz transmission was created for identifying hidden objects. Using a radiation of 0.12 THz, the shape of the glue-jar in the box was clearly visible (see Figure 12b). Furthermore, it was possible to detect not only the shape of the glue-jar but also the position of the amount of the glue inside the jar. Another key feature of the THz detector is its stability. Differently from black phosphorus [92], SnSe_2_-based devices exhibited better stability, congruently with the ambient stability assessed in Figure 6 by surface-science techniques. The absence of noticeable modifications in the photocurrent at 0.12 THz radiation after a prolonged storage in air (extended up to one month, Figure 12a) corroborates the excellent stability of SnSe_2_-based optoelectronic devices.

## 4. Conclusions

Here, we elucidated the main features of chemical reactivity of SnSe_2_. While the stoichiometric single crystal is chemically inert to ambient gases, the presence of selenium vacancies drastically affects surface chemical reactivity. The SnSe_2_−x surface is transformed into SnO_2_-skin-terminated SnSe_2_, with a thickness of the SnO_2_ skin estimated to be sub-nanometric. 

Interestingly, while the self-assembled SnO_2_/SnSe_2-x_ heterostructure is an exceptional platform for detecting chemical species, as demonstrated for H_2_, H_2_O, NO_2_, NH_3_, and HS_2_, the pristine SnSe_2_ is unable to detect the same species. Thus, our results highlight the pivotal role of Se vacancies in metal dichalcogenides, which can transform the system from ambient-stable to an ultrasensitive gas sensor by tuning the stoichiometry. 

Sensitivities of 0.43, 2.13, 0.11, and 1.06 [ppm]^−1^ were reported for the detection of H_2_, H_2_S, NH_3_, and NO_2,_ respectively. The corresponding limit of detection is 5 ppm, 10 ppb, 250 ppb, and 400 ppb for H_2_, H_2_S, NH_3_, and NO_2,_ respectively.

Moreover, SnSe_2_ is particularly suitable for THz photodetection, based on hot electrons. The response speed and the reactivity of the device are significantly improved thanks to the short channel, which exploits the localization of the electrostatic field and the high thermoelectric value of SnSe_2_. Furthermore, the device has excellent stability, even when the uncapped active channel is exposed to air for long periods, thanks to the exceptional chemical inertness of the single stoichiometric SnSe_2_ crystals. Accordingly, SnSe_2_-based photodetectors represent suitable and promising candidates for imaging applications for homeland security and quality controls. 

## Figures and Tables

**Table 1:** Differential enthalpy ΔHads and differential Gibbs free energy ΔG for physisorption at room temperature and differential enthalpy of decomposition ΔH_dec_ for molecular oxygen and water on the surface of bulk samples of SnSe_2_, SnSe_1.88_, and SnSe. For the decomposition of oxygen, we also report, in parenthesis, the differential enthalpy for the formation of an oxygenated surface and a SnO_2_-like layer.

Surface	Adsorbant	Physisorption		Decomposition
		ΔH_ads_ (kJ/mol)	ΔG (kJ/mol)	ΔH_dec_ (kJ/mol)
SnSe_2_	O_2_	−17.5	−3.2	−42.3 (−161.6/~ −40.2)
	H_2_O	−13.3	+18.0	+220.9
SnSe_1.88_	O_2_	−37.6	−26.3	−135.7 (−99.1/−406.7)
	H_2_O	−27.9	+3.4	+175.6
SnSe	O_2_	−11.6	−0.2	−236.1 (−323.1/+95.4)
	H_2_O	−8.1	+23.2	+82.2
SnO_2_ skin	H_2_O	−119.7	−106.7	−121.3

**Table 2:** Differential enthalpy (ΔH_phys_) and Gibbs free energy (ΔG) at an operational temperature of 200 °C for the physisorption of molecular oxygen at the surface of bulk, bilayer, and monolayer SnSe_2_ and SnSe_1.88_. The differential enthalphy for oxygen decomposition (ΔH_dec_) is also reported. All energies are expressed in kJ/mol.

System	Surface	ΔH_phys_ (kJ/mol)	ΔG_phys_ (kJ/mol)	ΔH_dec_ (kJ/mol)
Bulk	SnSe_2_ SnSe_1.88_	−17.5 −37.6	+5.24 −14.88	−42.3 −135.7
Bilayer	SnSe_2_ SnSe_1.88_	+38.9 −47.6	+61.6 −24.9	−76.3 −115.5
Monolayer	SnSe_2_ SnSe_1.88_	+53.1 −59.3	+75.8 −36.6	−56.3 +183.5

**Table 3:** Differential Gibbs free energies ΔG of physisorption at 200 °C and corresponding values of transferred charge for various combination of considered substrates and analytes

Substrate	Analyte	ΔG (kJ/mol)	Δe^−^
Bulk SnSe_2_ (SnSe_1.88_)	H_2_ H_2_O H_2_S NH_3_ NO_2_	+6.79 (−0.11) +45.63 (−30.97) +38.70 (−21.32) +24.50 (−12.10) +58.87 (−36.75)	+0.09 (+0.10) −0.17 (−0.20) −0.12 (−0.14) +0.06 (+0.12) −0.07 (−0.10)
Bilayer SnSe_2_ (SnSe_1.88_)	H_2_ H_2_O H_2_S NH_3_ NO_2_	+8.91 (−0.10) +41.23 (−26.8) +36.14 (−29.65) +18.91 (−10.01) +46.75 (−25.44)	+0.08 (+0.10) −0.15 (−0.18) −0.10 (−0.13) +0.10 (+0.15) −0.10 (−0.11)
Monolayer SnSe_2_ (SnSe_1.88_)	H_2_ H_2_O H_2_S NH_3_ NO_2_	+2.89 (−4.63) +28.97 (−7.11) +18.35 (−0.12) −4.91 (−12.10) +0.12 (−3.45)	+0.10 (+0.09) −0.15 (−0.20) −0.12 (−0.09) +0.16 (+0.15) −0.09 (−0.10)
SnO_2_/SnSe_2_	H_2_ H_2_O H_2_S NH_3_ NO_2_	−135.61 −60.80 −71.28 −96.20 −127.35	+0.15 −0.43 −0.36 +0.44 −0.25

**Table 4:** Sensing of H_2_, H_2_S, NH_3_, and NO_2_ for SnSe_2_- and SnO_2_-based systems. The response obtained at the respective operational temperature and gas concentration is reported. RT means room temperature.

Gas	Sensing Materials	OperationalTemperature (°C)	Concentration (ppm)	Response	Reference
H_2_	SnO_2_/SnSe_2-x_	150	100	3	[68]
H_2_	SnO_2_	150	1000	5.5	[75]
H_2_S	SnO_2_	100	10	1–6	[76]
H_2_S	SnSe_2_	RT	10	10–15	[77]
H_2_S	SnO_2_/SnSe_2_	RT	10	32	[70]
H_2_S	SnO_2_	RT	50	33	[78]
NH_3_	SnO_2_/SnSe_2_	RT	100	2	[70]
NH_3_	SnSe_2_	RT	40	2.7	[79]
NH_3_	Au-SnSe_2_	RT	5	5.3	[80]
NO_2_	SnO_2_/SnSe_2_	RT	10	3.5	[70]
NO_2_	SnO_2_/SnSe_2-x_	150	1	3.2	[68]
NO_2_	SnSe_2_	RT	1	6	[79]
NO_2_	SnSe_2_	RT	5	112	[81]
NO_2_	SnSe_2_/SnSe	RT	1	75	[82]
NO_2_	Au/SnSe_2_	130	8	3	[83]
NO_2_	Pt-SnSe_2_	130	8	3.9	[83]
NO_2_	SnSe/SnSe_2_	RT	5	12	[84]
NO_2_	SnSe_2_	RT	8	1.4	[85]
NO_2_	SnO_2_	100	10	1	[76]

**Table 5:** Sensitivity to H_2_, H_2_S, NH_3_, and NO_2_ for SnSe_2_-based sensors, as well as their limit of detection.

Gas	Sensitivity [ppm]^−1^	Limit of Detection
H_2_	0.43 ± 0.02 [68]	5 ppm at 150 °C [68]
H_2_S	2.13 ± 0.01 [77]	10 ppb at RT [70]
NH_3_	0.11 ± 0.01 [80]	250 ppb at RT [80]
NO_2_	1.06 ± 0.03 [68]	400 ppb at 150 °C [68]

## Supplementary Materials

The following supporting information can be downloaded at: https://www.mdpi.com/article/10.3390/ma15031154/s1, File S1: Theoretical Methods; File S2: Fabrication process and measurements of devices.
