# Supplementary material for: Tin Diselenide (SnSe2) Van der Waals Semiconductor: Surface Chemical Reactivity, Ambient Stability, Chemical and Optical Sensors
Source: Materials (Basel). 2022 Feb 2;15(3):1154. doi: 10.3390/ma15031154 (PMC8838464; doi:10.3390/ma15031154)
Supplement: Supplementary file 1 [file materials-15-01154-s001.zip › materials-1522459-supplementary.pdf]

Supplementary Materials

Tin Diselenide ($SnSe_2$) Van der Waals Semiconductor: Surface Chemical Reactivity, Ambient Stability, Chemical and Optical Sensors

Gianluca D'Olimpio [1], Daniel Farias [2,3,4,*], Chia-Nung Kuo [5,6], Luca Ottaviano [1,7], Chin Shan Lue [5,6], Danil W. Boukhvalov [8,9,*] and Antonio Politano [1,10,*]

[1] Department of Physical and Chemical Sciences, University of L'Aquila, via Vetoio, 67100 L'Aquila (AQ), Italy
[2] Departamento de Física de la Materia Condensada, Universidad Autónoma de Madrid, 28049 Madrid, Spain
[3] Instituto "Nicolás Cabrera", 28049 Madrid, Spain
[4] Condensed Matter Physics Center (IFIMAC), 28049 Madrid, Spain
[5] Department of Physics, National Cheng Kung University, 1 Ta-Hsueh Road, 70101 Tainan, Taiwan
[6] Taiwan Consortium of Emergent Crystalline Materials, Ministry of Science and Technology, Taipei 10601, Taiwan
[7] CNR-SPIN UoS L'Aquila, Via Vetoio, I-67100 L'Aquila, Italy
[8] College of Science, Institute of Materials Physics and Chemistry, Nanjing Forestry University, Nanjing 210037, P. R. China
[9] Theoretical Physics and Applied Mathematics Department, Ural Federal University, Mira Street 19, 620002 Ekaterinburg, Russia
[10] CNR-IMM Istituto per la Microelettronica e Microsistemi, VIII strada 5, I-95121 Catania, Italy

* Correspondence: daniel.farias@uam.es (D.F.); danil@njfu.edu.cn (D.W.B.); antonio.politano@univaq.it (A.P.)

S1. Theoretical Methods

Modelling of the atomic structure and energetics of gas adsorption on Pt_3Te_4 was carried out using the QUANTUM-ESPRESSO code [1] and the GGA−PBE functional with van der Waals (vdW) corrections, feasible for the studying of the adsorption of molecules on surfaces [2,3]. Energy cutoffs of 25 and 400 Ry for the plane-wave expansion of the wave functions and the charge density, respectively, and the 4 × 4 × 3 Monkhorst-Pack k-point grid for the Brillouin sampling were used [4]. For the modeling of the surface, we used slab of two Pt_3Te_4 layers each of these layers contain $PtTe_2$ and Pt_2Te_2 layers (see Figure 1). Note that in the slab of any number of Pt_3Te_4 layers on one surface will be Pt_2Te_2 layer and on opposite side $PtTe_2$. Moreover, we also considered the presence of the Te vacancies in top layer. To imitate contribution from rigid subsurface area of bulk crystals in the modeling of the surfaces, we performed optimization of the only atomic positions. In order to take into account the contribution from flexibility of nanosheets, we performed optimization of both atomic positions and lattice parameters. The size of vacuum layers between the slabs have been set as 7*c* lattice parameters considered systems. Thus, the value of vacuum layer is about 5 nm that is corresponding with 15 van der Waals radii (0.35 nm) and therefore we can claim the absence of interaction of considered molecules with another slab within periodic boundary conditions.

The enthalpies of physical adsorption were calculated by the standard formula:

$$\Delta H_{phys} = [E_{host+mol} - (E_{host} + E_{mol})] \qquad (1)$$

Here, E_{host} is the total energy of the surface before adsorption, and E_{mol} is the energy of the single molecules of considered species in empty box. In the case of water adsorption, we only considered adsorption from the gaseous phase. Energy of chemical adsorption is defined as difference between the total energy of the system

after and before decomposition of physically adsorbed molecule. For the case of physical adsorption, we also evaluated differential Gibbs free energy the formula

$\Delta G = \Delta H - T\Delta S$ (2)

where T is the temperature and ΔS is the change of entropy after formation molecule–substrate noncovalent bond, which was estimated similar to the gas → liquid transition and hence can be evaluated by the standard formula

$\Delta S = \Delta H_{vap}/T$ (3)

where ΔH_{vap} is the empirical enthalpy of vaporization.

S2. Fabrication process and measurements of devices

The $SnSe_2$-based photodetectors are fabricated starting from mechanically exfoliated flakes of $SnSe_2$ on top of silicon wafer capped with a thin oxide layer SiO_2 (300 nm). Ultraviolet lithography was used to define the source/drain patterns, and Au/Cr (60/5 nm) film was deposited using an electron beam evaporator (Ulvac Ei-5Z). Finally, we obtained contact terminals by lift-off to selectively remove the unprotected areas by a lithographic mask.

The THz radiations at different frequencies were provided by multiplier of electronic source. Power density was calibrated by a Golay cell. Our THz photoelectric measurements were also performed at room temperature in ambient conditions. When one electrode open circuit is configured, the other electrode is kept grounded at the same time. The photoresponse was collected by a lock-in amplifier (LIA) or an oscilloscope after a low-noise voltage preamplifier. The value of I_{ph} can be get from the photo signal on the LIA via the relation $I_{ph} = 2.2LIAG_n^{-1}$, where G_n is gain factor and 2.2 accounts for the sine wave Fourier component.

References

1. Giannozzi, P.; Baroni, S.; Bonini, N.; Calandra, M.; Car, R.; Cavazzoni, C.; Ceresoli, D.; Chiarotti, G.L.; Cococcioni, M.; Dabo, I. QUANTUM ESPRESSO: a modular and open-source software project for quantum simulations of materials. *Journal of physics: Condensed matter* **2009**, *21*, 395502.
2. Perdew, J.P.; Burke, K.; Ernzerhof, M. Generalized Gradient Approximation Made Simple. *Phys. Rev. Lett.* **1996**, *77*, 3865-3868.
3. Barone, V.; Casarin, M.; Forrer, D.; Pavone, M.; Sambi, M.; Vittadini, A. Role and effective treatment of dispersive forces in materials: Polyethylene and graphite crystals as test cases. *Journal of Computational Chemistry* **2009**, *30*, 934-939, doi:10.1002/jcc.21112.
4. Monkhorst, H.J.; Pack, J.D. Special points for Brillouin-zone integrations. *Phys. Rev. B* **1976**, *13*, 5188-5192.
5. Greeley, J.; Jaramillo, T.F.; Bonde, J.; Chorkendorff, I.; Nørskov, J.K. Computational high-throughput screening of electrocatalytic materials for hydrogen evolution. *Nature materials* **2006**, *5*, 909-913.
6. Tang, Y.; Allen, B.L.; Kauffman, D.R.; Star, A. Electrocatalytic activity of nitrogen-doped carbon nanotube cups. *Journal of the American Chemical Society* **2009**, *131*, 13200-13201.
